# Augmented feedback for manual wheelchair propulsion technique training in a virtual reality simulator

**DOI:** 10.1186/s12984-021-00936-x

**Published:** 2021-09-21

**Authors:** Hui Yan, Philippe S. Archambault

**Affiliations:** 1 Integrated Program in Neuroscience, McGill University, Montreal, Canada; 2 Center of Interdisciplinary Research in Rehabilitation of Greater Montreal (CRIR), Montreal, Canada; 3 School of Physical and Occupational Therapy, McGill University, Montreal, Canada

**Keywords:** Manual wheelchair, Propulsion training, Virtual reality, Augmented feedback

## Abstract

**Background:**

Motor learning of appropriate manual wheelchair propulsion is critical, as incorrect technique elevates risk for upper extremity pain. Virtual reality simulators allow users to practice this complex task in a safe and realistic environment. Additionally, augmented feedback (AF) may be provided in order to optimize learning. The purpose of this study was to investigate the effects of providing AF with various delivery schedules on motor learning and transfer of this skill to over-ground propulsion.

**Methods:**

Thirty healthy young adults were randomly assigned to three groups. During a virtual reality propulsion training session, the high-frequency AF group received AF in the form of knowledge of performance throughout all propulsion training; the faded AF group received this AF in a faded schedule (high relative frequency of AF early in practice, with relative frequency of AF provision diminishing throughout practice); and the control group underwent training with no AF. Propulsion assessments were performed at baseline and 48 h after practice in both virtual and real environments to measure retention and transfer, respectively.

**Results:**

Compared to the control group, both feedback groups exhibited significant improvements in contact angle and push frequency in both environments after training. Small, non-significant between-group differences were also found between the high-frequency and faded feedback groups.

**Conclusion:**

Virtual reality training is an effective learning intervention for acquisition, retention, and transfer of appropriate manual wheelchair propulsion technique when such training includes AF regarding propulsion biomechanics.

## Background

For individuals with mobility impairments, using a manual wheelchair (MWC) is an effective and accessible form of both mobility and aerobic exercise [1, 2]. However, largely due to the highly repetitive and strenuous nature of MWC propulsion, a significant proportion of long-term users develop pain and injury in the upper extremities. As many as 73% of users experience shoulder pain and up to 55% suffer from carpal tunnel syndrome [3–7]. Prevention of such injuries may be achieved through learning safe and efficient propulsion technique. In new MWC users, propulsion is a novel motor skill that must be acquired through motor learning principles as this ideal technique is far from intuitive.

Ergonomics and propulsion biomechanics research have identified several indices of propulsion quality and many bear complex relationships with risk for upper limb pain and injury. For example, push frequency, defined as the number of pushes per second, has been positively correlated with shoulder pain as well as impaired median nerve function [8, 9]. Sawatzky et al. [10] recommend that during steady-state MWC propulsion, this measure should be maintained as one push/second or less. Contact angle, defined as the angle over which the hand is in contact with the MWC pushrim, should be maintained between 85° and 100° [10]. A large contact angle promotes lower push frequency, smoother pushes, and lower rate of rise of force [11–13]. However, absolutely maximizing contact angle may also engender dangerous consequences as this goal promotes movement at the extremes of upper limb ranges of motion and repeatedly places joints at awkward and potentially injurious positions [14, 15].

Additionally, the clinical practice guidelines from the Consortium for Spinal Cord Medicine recommend that MWC users use long, smooth pushes which limit the amount of peak force applied to the pushrim. The guidelines also recommend the adoption of a semi-circular pattern, in which the hand follows the path of the pushrim, drifting below the rim during the recovery phase of the push [15]. The semi-circular pattern is specifically preferred because it has been associated with greater push time to recovery time ratio, lower push frequency, low joint acceleration, and minimal abrupt changes in hand direction [16–18]. In summary, the ideal technique appears to involve a semi-circular propulsion pattern with low push frequency, relatively large contact angle, and limitations on force. However, this pattern does not appear to be naturally intuitive for most new MWC users [11, 19, 20].

Evidently, safe and efficient MWC propulsion technique is a complex, novel motor skill that must be acquired using motor learning principles. However, new users are afforded insufficient time for specific propulsion training with a clinician during rehabilitation. Clinicians in Canadian rehabilitation centers are able to commit, on average, only 1–4 h to all MWC training per patient [21]. Virtual reality (VR) systems and simulators may serve as a potential solution to this problem, while also offering unique advantages such as safety, motivation, and increased practice time with fewer clinicians required [22, 23]. The current evidence also suggests that training protocols using VR-based simulators are valid and effective for facilitating positive changes in manual, as well as power wheelchair performance [24–26].

VR-based training also presents as a unique opportunity to measure and provide feedback to the user about their performance in real time. Specifically, augmented feedback (AF) is defined as information that is provided in addition to intrinsic sensory information that is naturally perceived during practice [27, 28]. A number of studies support the contention that AF about propulsion biomechanics can effectively and reliably produce desirable change in contact angle and push frequency [14, 29–32]. However, the effect of AF on improvement of kinetic parameters such as peak force, fraction of effective force, and power output is less consistent [32–34]. Notably, all of these studies have been performed on wheelchair ergometers or treadmills without VR, and only two utilized delayed retention tests [29, 30]. Further, only one of these studies evaluated transfer of acquired skill from ergometer or treadmill to over-ground propulsion [29].

During VR training, AF may also be manipulated in modality, content, and timing, among other factors in order to facilitate error detection and learning. For example, feedback may be provided concurrently or terminally (i.e., during or after a trial), as well as visually, audibly, or haptically. Other elements of AF that may be varied are its relative frequency and delivery schedule. In contrast to predictions based on traditional motor learning theories, the guidance hypothesis asserts that feedback is useful early in practice but detrimental when relied upon for long periods of time [35]. In a faded feedback schedule, AF is provided at high frequency early in practice and is gradually diminished as practice progresses. The guidance hypothesis predicts that such a faded feedback schedule may be an ideal approach to learning as it captures the benefits of AF by guiding learners to correct movement patterns early in practice while avoiding dependence [36, 37]. The guidance hypothesis has been robustly verified for training discrete, laboratory tasks such as manipulating a lever or producing target force waveforms [38–40]. However, it has not been confidently verified in more complex, ecological, and continuous tasks such as MWC propulsion.

When considering acquisition and learning of any motor task, it is imperative to emphasize the critical distinction between motor performance and motor learning. Motor learning refers specifically to long-term, relatively permanent changes in movement execution or capability. Meanwhile, transient improvements in motor performance may occur as a result of specific practice conditions [27]. For this reason, it is critical to administer delayed retention tests after sufficient time without practice in order to identify and measure true motor learning [41]. Furthermore, when training takes place in a controlled setting such as with a laboratory ergometer or in a VR simulator, it is also critical to consider whether acquired skills and techniques have real-world benefits. Wheelchair propulsion quality must also be measured over-ground before and after VR training in order to confirm that learning is not exclusively limited to simulator propulsion.

The purpose of the current study was to examine the effects of providing AF as well as its delivery schedule on motor learning, retention, and transfer of MWC propulsion technique as a novel motor skill. Specifically, this study sought to (1) compare the relative benefits of providing AF during training to conditions without AF, (2) compare a faded schedule to a high frequency schedule of AF, and (3) determine the transferability of technique acquired in a VR simulator.

## Methods

### Participants

Thirty healthy participants aged 18–35 years from the Greater Montreal Area were recruited for this study. In order to examine motor learning of MWC propulsion as a novel skill, participants were excluded if they had any significant previous experience using a wheelchair. Individuals with any self-reported sensory or motor difficulties which could hinder the use of a MWC or VR simulator were also excluded. The Montreal Cognitive Assessment (MoCA) was used to assess participants’ cognitive health. Those with cognitive impairments (cutoff: < 26/30) which could have hindered MWC and simulator use were also excluded from the study. The 30 participants were randomly assigned, using simple randomization procedures, to one of three groups; a high frequency feedback group (HF-F), a faded feedback group (F-F), and a control group.

This study took place at the Jewish Rehabilitation Hospital in Laval, QC and was approved by the ethics committee of the Centre for Interdisciplinary Research in Rehabilitation of Greater Montreal (CRIR). All participants provided informed consent upon recruitment.

### Experiment design

**Fig. 1:**
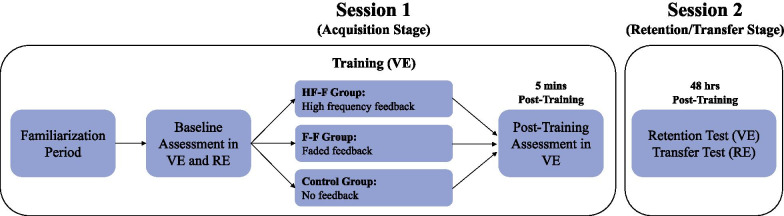
Experiment design—summary (*VE* virtual environment, *RE* real environment, *HF-F* high-frequency augmented feedback group, *F-F* faded feedback augmented group)

The experiment is designed as a randomized control trial. As a motor learning study, this experiment consisted of an acquisition stage and a retention/transfer stage, 48 h apart (Fig. 1). For each participant, the acquisition stage required approximately 2 h in total, and the retention/transfer stage required approximately 30 min. During the acquisition stage, all participants were introduced to the VR simulator and were provided with instructions about appropriate propulsion technique using statements from clinical practice guidelines [15]. Participants were then allowed a familiarization period which provided participants with 5 min to become comfortable maneuvering the MWC in the simulator as well as 5 min over-ground. During this familiarization period, we ensured that participants could comfortably maintain propulsion velocity above 1.06 m/s in both settings, which is the minimum recommended velocity for safe community navigation [42]. This was done by allowing participants to practice several times in both environments while measuring average velocity using instrumented wheels attached to the MWC. Next, baseline propulsion assessments were conducted in both a virtual (VE) and real environment (RE).

Following the initial familiarization and assessment, all participants underwent propulsion training in the VR simulator. For all groups, training consisted of 18 consecutive practice trials which required participants to propel down a straight virtual sidewalk that contained realistic and challenging obstacles, interspersed with straight-line, no obstacle segments. Each practice trial lasted approximately 1–2 min, depending on how long each participant required to navigate the full sidewalk. Between practice trials, participants were offered a short break, if needed. For the HF-F group, AF regarding propulsion technique was presented for all steady-state propulsion segments. The F-F group received this AF in a faded schedule that gradually diminished in frequency throughout training. The control group underwent the same practice trials, but with no AF during any of the trials.

Five minutes following practice, a post-training propulsion assessment was performed in the VE only. During the retention/transfer stage, 48 h following training, propulsion assessments were repeated in the VE and RE as retention and transfer tests, respectively.

### miWe simulator

**Fig. 2:**
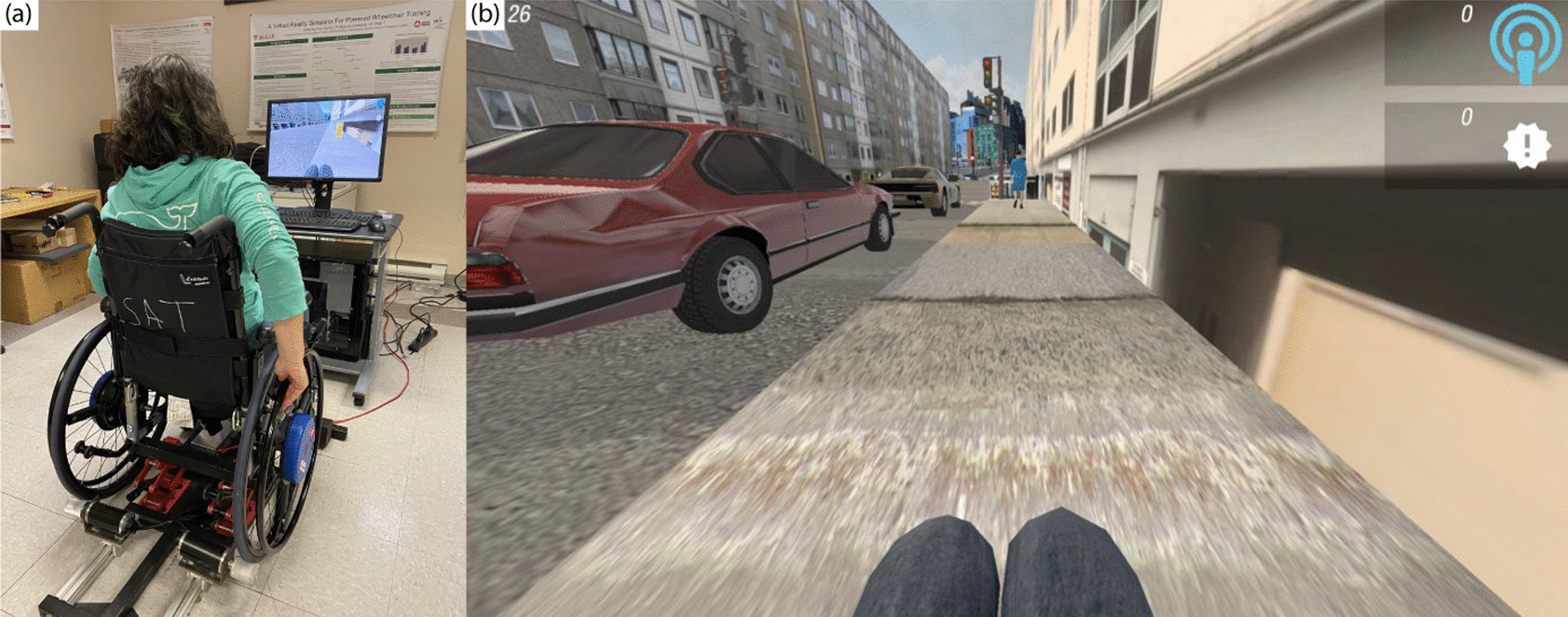
**a** miWe simulator setup, **b** miWe virtual environment presented on screen

The VR simulator used in this study was the MWC version of the McGill Immersive Wheelchair Simulator (miWe) (McGill University, Montreal, QC) [43]. Using this system, the learner propelled a MWC placed on a haptic platform in order to interact with elements in a VE, which was presented on a computer monitor placed at eye level (Fig. 2a). The motors of the haptic platform applied forces to the MWC wheels to simulate real world forces and dynamics. This system used a non-immersive VR approach in order to limit discomfort and nausea as well as to facilitate a low-cost and portable design.

An “infinite sidewalk” virtual scenario was designed for practicing propulsion as well as other skills by allowing the user to traverse a realistic sidewalk for a specified amount of time (Fig. 2b). The system allowed for careful manipulation of VE characteristics such as the presence of obstacles including ramps, slopes, signs, pedestrians, and street crossings in the infinite sidewalk. Finally, for this study, the infinite sidewalk scenario was further developed to allow for provision of AF during training or gameplay. The biomechanical variables of contact angle, push frequency, velocity, and push time were measured in real time by the haptic platform and this information was provided to the user in a feedback interface presented on screen.

### Propulsion assessments

**Fig. 3:**
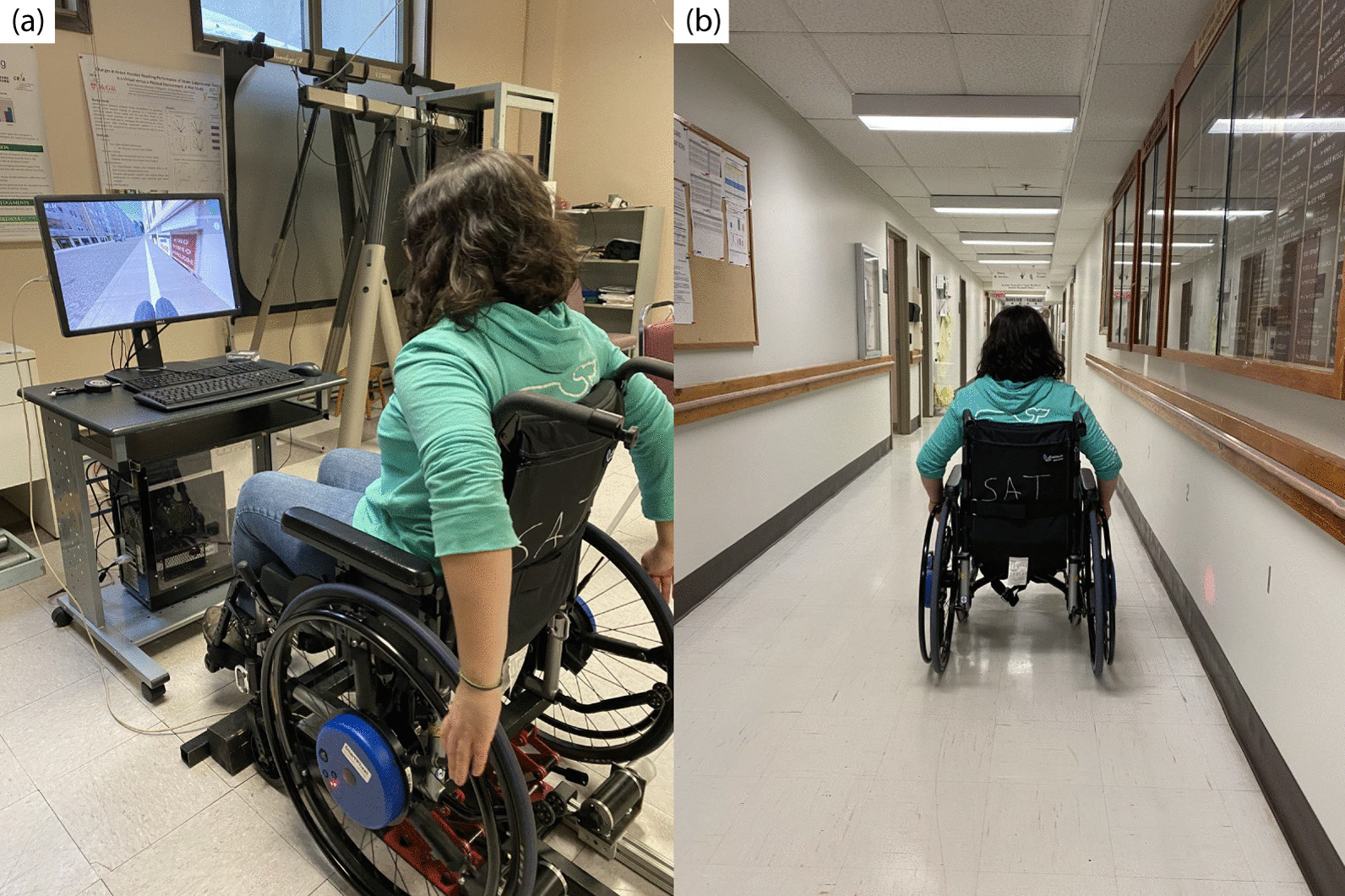
**a** VE propulsion assessment, **b** RE propulsion assessment

Participants performed propulsion assessments in the VE at baseline, 5 min post-training (short-term), and 48 h post-training (long-term). Propulsion assessments in the RE were also administered at baseline and 48 h post-training (long-term). For all assessments, participants were asked to propel down a long, straight, empty path for 60 s, while maintaining a minimum velocity of 1.06 m/s. In the VE, a segment of the infinite sidewalk containing no obstacles was used for these assessments (Fig. 3a). In the RE, assessments were conducted in an empty hallway (Fig. 3b).

During all assessments, contact angle and push frequency were taken as indices of propulsion quality. These outcomes were measured using a SMART^Wheel^ (Three Rivers Holdings LLC, Meza, AZ) an instrumented wheel mounted to a standard MWC [44, 45]. Velocity was also monitored during each assessment to ensure that the minimum threshold was met. If the average velocity taken throughout any 60-s assessment in the VE or RE did not meet the minimum velocity, the participant was asked to repeat the assessment in order to achieve this minimal average velocity. Most of the participants were able to perform the assessment with this velocity on the first attempt, and none required more than one additional attempt. The short-term post-training assessment was used only to observe initial changes in motor performance due to practice. Meanwhile, the long-term assessments in the VE and RE were administered as retention and transfer assessments respectively in order to identify the amount of motor learning and effective transfer of learning to real-world propulsion.

### Practice trials

**Fig. 4:**
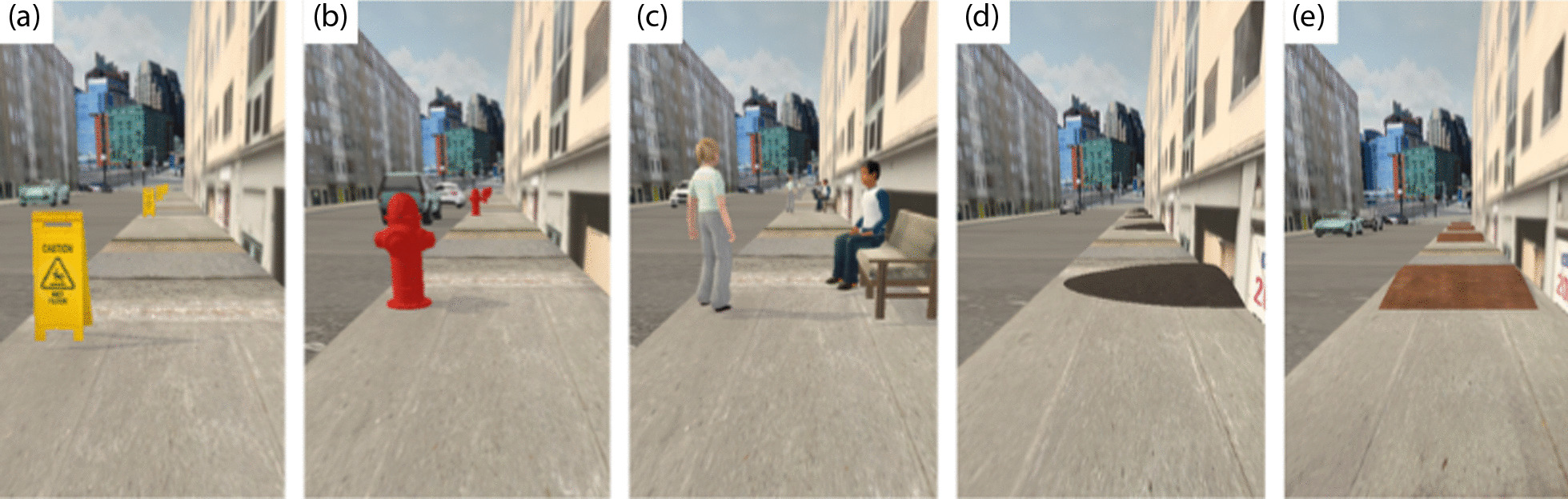
Examples of obstacles. **a** Left sign, **b** Hydrant, **c** Bench conversation, **d** Side Slope, **e** Straight Slope. Other available obstacles include Fallen Sign, Vending Machine, Street Crossing, and more

The miWe simulator allows users, clinicians, or investigators to incorporate a variety of realistic obstacles into the sidewalk that is traversed during training (Fig. 4) [43]. Such obstacles have been identified by MWC users as relevant challenges in daily life. In this study, during VR training, practice trials consisted of three empty “no obstacle” (N.O.) sections of sidewalk separated by a random selection of obstacles (Table 1) such as side slopes, benches, street crossings, and others (Fig. 4). The presence of obstacles during training is favourable for reinforcing motor learning. Not only do they increase overall task realism and difficulty, they may be modified between trials to introduce variability of practice, a factor that is remarkably beneficial for both retention and transfer of motor skills including wheelchair propulsion [27, 46–49]. Additionally, random scheduling of various tasks and task parameters during practice is beneficial for both retention and transfer of skill [50–52].

**Table 1:** Practice trials

Sidewalk blocks					
No obstacle	Random obstacle	No obstacle	Random obstacle	No obstacle	Random obstacle

### Augmented feedback

While there are many outcome measures that can be used to describe propulsion quality, in this study, contact angle and push frequency were chosen for AF for several reasons. Firstly, for these two outcome measures, there currently exist specific, quantitative recommendations and guidelines in the literature [10, 15]. Based on propulsion studies, it has been recommended to maintain contact angle between 85° and 100° and stroke frequency below 1 push/s during steady-state propulsion [10]. Secondly, existing evidence suggests that unlike kinetic variables such as peak force or fraction of effective force, the temporal variables of contact angle and push frequency are easy for users to understand and modify when AF is presented [14, 29, 32, 53]. Finally, the outcome measures of contact angle and push frequency are easy to measure using low-cost methods and equipment. Throughout the development of the miWe simulator, affordability and portability have been fundamental priorities. While kinetic measures would require equipment such as instrumented wheels or force sensors, contact angle and push frequency may be measured and presented as AF in real-time using the simulator itself.

**Fig. 5:**
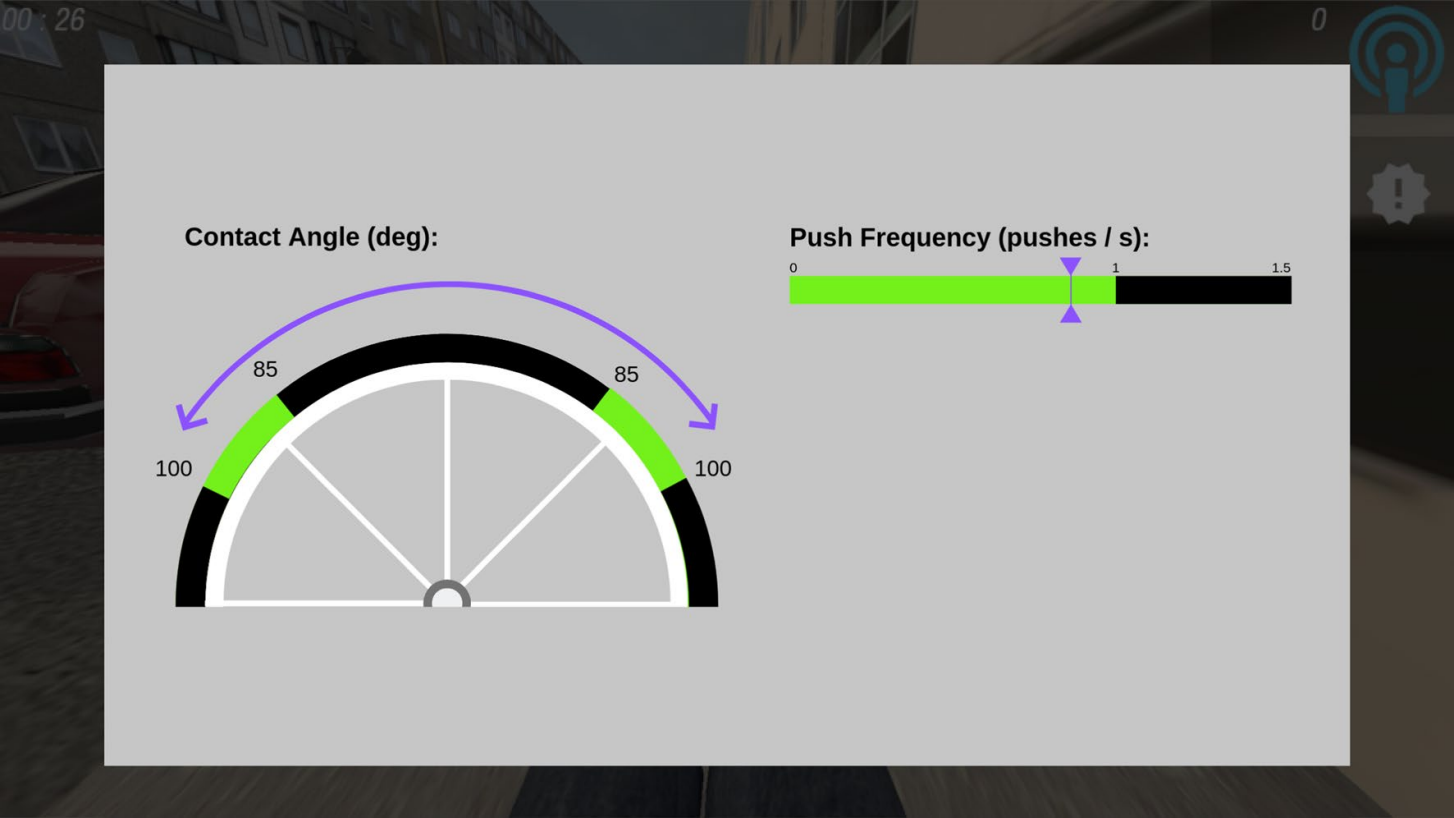
Augmented feedback during practice trials. Green areas represent the range of acceptable contact angle and push frequency. Purple markers show the user’s average value during the previous block

AF presented in this study was visual, terminal, summary feedback (Fig. 5). Average contact angle and push frequency for all pushes that occurred in full during a given N.O. segment were shown for 10 s after that segment was completed. Three different practice trial types were designed: high-frequency augmented feedback (HF) trials, during which AF was provided after each of the three N.O blocks; low-frequency augmented feedback (LF) trials, for which this information was provided after the third N.O. block; and during no augmented feedback (NF) trials, which contained no AF presentation. The HF-F group performed 18 HF practice trials; the F-F group underwent six HF practice trials, followed by six LF practice trials, followed by six NF practice trials; the control group performed 18 NF practice trials (Table 2).

**Table 2:** Practice and feedback schedule by group

Trial	1	2	3	4	5	6	7	8	9	10	11	12	13	14	15	16	17	18
HF-F Group	HF	HF	HF	HF	HF	HF	HF	HF	HF	HF	HF	HF	HF	HF	HF	HF	HF	HF
F-F Group	HF	HF	HF	HF	HF	HF	LF	LF	LF	LF	LF	LF	NF	NF	NF	NF	NF	NF
Control Group	NF	NF	NF	NF	NF	NF	NF	NF	NF	NF	NF	NF	NF	NF	NF	NF	NF	NF

*HF* high frequency augmented feedback, *LF* low frequency augmented feedback, *NF* no augmented feedback

In addition, participants in all groups received verbal reminders regarding the performance measures of velocity and propulsion pattern. Specifically, if an average velocity of 1.06 m/s was not reached for a given N.O. block, an investigator would remind the participant to increase velocity on the next N.O. block. Additionally, an investigator visually monitored the participant’s propulsion pattern during obstacle-free propulsion and reminded them to use the semi-circular pattern if propulsion deviated from this technique.

### Data analysis

Mean contact angle and push frequency were calculated for each group’s baseline, short-term, and long-term post-training assessments in both the VE and RE. Additionally, since there exists both a minimum and maximum to the target range for contact angle, performance was additionally evaluated by calculating the proportion of pushes during each assessment that fell within the target range of 85°–100°. All three outcomes were tested for normality using the Shapiro–Wilk test. Contact angle and push frequency were found to be normally distributed, but the proportion of contact angles in range was not. As such, in order to observe differences between baseline and short-term/retention/transfer as well as between groups, a repeated, mixed model analysis of variance was run for each of mean contact angle and push frequency with one between-subject (feedback schedule: HF-F, F-F or control) and two within-subject factors (time: baseline or retention/transfer; and environment: VE or RE). A mixed model analysis of variance was chosen as it involves a model for the error variance, leading to more efficient estimates and powerful tests [54]. An unstructured variance matrix was used as this offered the best fit with the data. Non-parametric tests were used to observe differences in proportion of contact angles in range between baseline and short-term/retention/transfer as well as between groups. For each variable, specific custom hypotheses were tested in order to address the aims of this study: (1) comparison between baseline and short-term assessment for each group in the VE; (2) comparison between baseline and retention assessment for each group in the VE; (3) comparison between baseline and transfer assessment for each group in the RE; (4) comparison between groups at short-term assessment in the VE; (5) comparison between groups at long-term retention assessment in the VE; and (6) comparison between groups at long-term transfer assessment in the RE.

## Results

### Mean contact angle

For the measure of mean contact angle, tests of fixed effects confirmed that both group (F_2,54_ = 9.1, p < 0.001) and time (F_1,54_ = 83.5, p < 0.001) each had a significant effect on the measure of mean contact angle. The effect of environment was not significant, however, the effect of the interaction between group, time, and environment was significant (F_7,69.4_ = 4.7, p < 0.001).

**Fig. 6:**
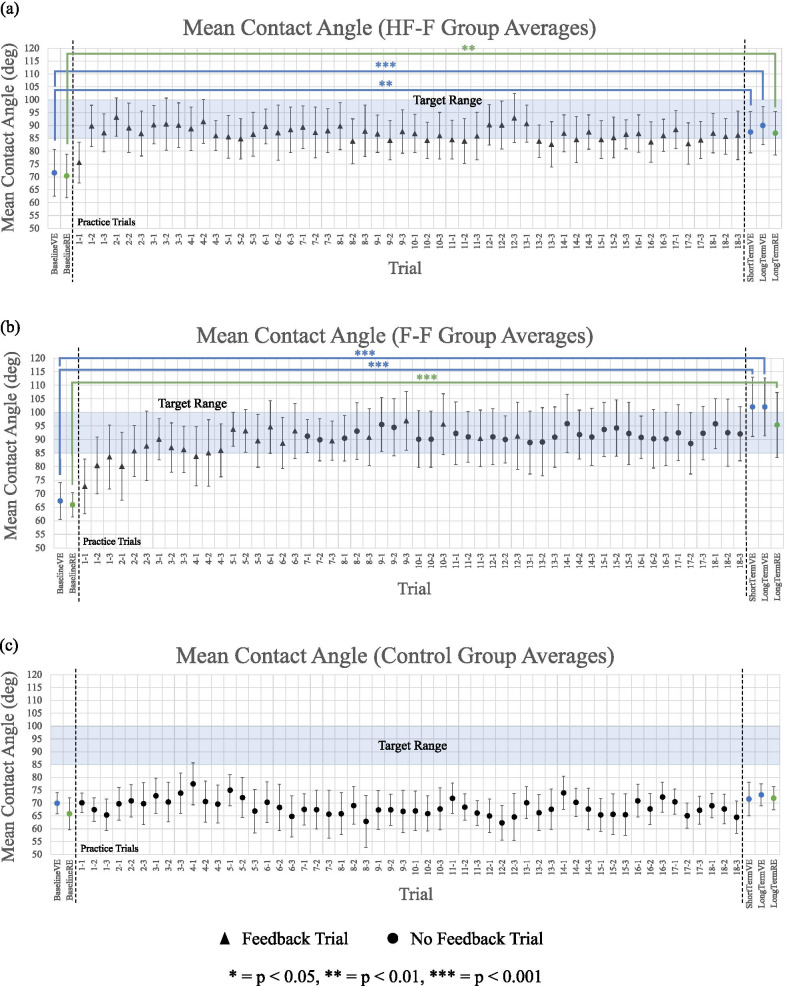
Mean contact angle (group average) for assessments (in VE and RE) and practice trial sequences for **a** HF-F group, **b** F-F group, and **c** control group. Each practice trial is split into three segments of straight-line, “no obstacle” sections of sidewalk, each separated by a random obstacle. Error bars represent 95% confidence intervals

Figure 6 displays group averages for mean contact angle in all assessments and practice trial segments for the HF-F group (Fig. 6a), the F-F group (Fig. 6b), and the control group (Fig. 6c). In the VE, the HF-F group improved mean contact angle from 71.6° to 87.4° (t_51.6_ = 2.7, p < 0.01; standard error (SE) = 5.7) from baseline to short-term assessment. This corresponded to an effect size of d = 0.8. This learning was also retained at long-term retention assessment. From baseline to retention assessment in the VE, the HF-F group significantly increased mean contact angle from 71.6° to 90.0° (t_54_ = 3.8, p < 0.001; SE = 4.8; d = 1.0). Further, the transfer test indicated that this group improved mean contact angle significantly from 70.4° to 87.0° in the RE (t_54_ = 3.4, p < 0.01; SE = 4.8; d = 0.9). In the VE, from baseline to short-term assessment, the F-F group also significantly increased this measure from 67.3° to 102.0° (t_51.6_ = 6.1, p < 0.001; SE = 5.7; d = 1.7). This group also retained this learning, increasing mean contact angle from 67.3° to 102.0° (t_54_ = 7.2, p < 0.001; SE = 4.8; d = 1.9) in the VE retention test. The F-F group also significantly improved from 65.9° to 95.4° in the RE (t_54_ = 6.1, p < 0.001; SE = 4.8; d = 1.7). Meanwhile, the control group saw no significant changes in mean contact angle before and after training in either environment.

**Fig. 7:**
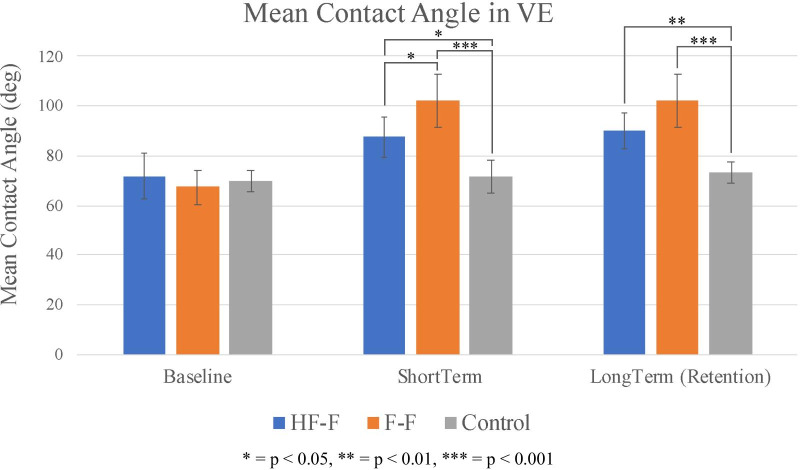
Mean contact angle (group average) for baseline, short-term, and long-term propulsion assessments in the virtual environment. Error bars represent 95% confidence intervals

Figure 7 shows mean contact angle for each group at baseline, short-term, long-term propulsion assessment in the VE. At the short-term assessment, differences were found between all groups. The HF-F group had significantly greater mean contact angle than the control group (t_27_ = 2.5, p < 0.05; SE = 6.3; d = 1.0). The mean contact angle of the F-F group was also significantly greater than that of the control group (t_27_ = 4.9, p < 0.001; SE = 6.3; d = 1.9). Finally, compared to the HF-F group, the F-F group had significantly greater mean contact angle (t_27_ = 2.3, p < 0.05; SE = 6.3; d = 0.9). At long-term retention assessment, group differences were found between each of the feedback groups and the control group. That is, a significant difference was seen between the HF-F group and the control group (t_54_ = 2.8, p < 0.01; SE = 6.1; d = 0.8). The F-F group also had significantly greater mean contact angle than the control group (t_54_ = 4.8, p < 0.001; SE = 6.1; d = 1.3). However, while the F-F group exhibited a larger mean contact angle than the HF-F group, this difference was not significant.

**Fig. 8:**
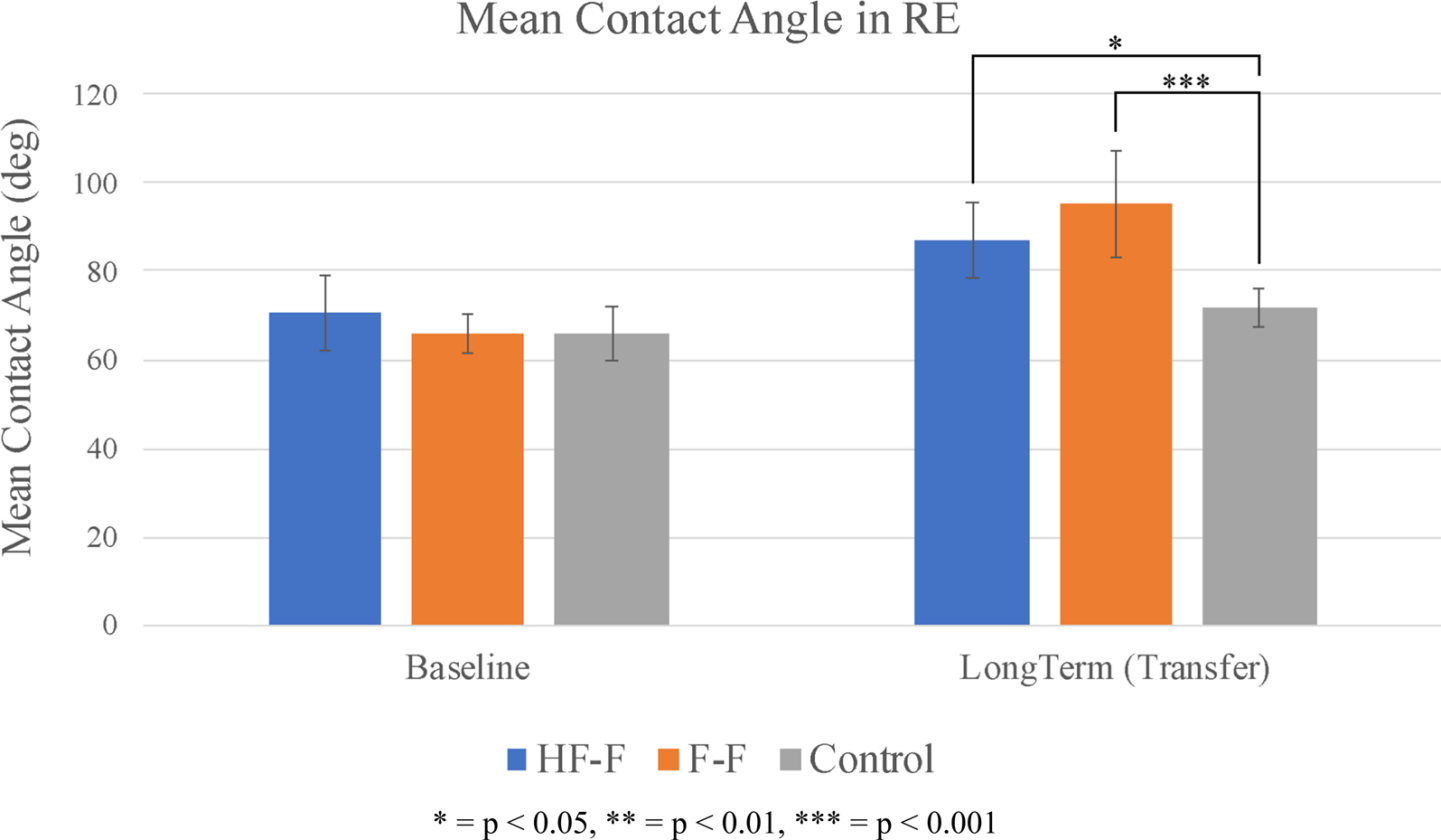
Mean contact angle (group average) for baseline and long-term propulsion assessments in the real environment. Error bars represent 95% confidence intervals

The mean contact angle for each group at baseline and 48 h post-training in the RE is shown in Fig. 8. At the long-term transfer assessment, dramatic differences were seen between the two feedback groups and the control group. Mean contact angle was significantly greater in the HF-F group compared to the control group (t_54_ = 2.5, p < 0.05; SE = 6.1; d = 0.7). Moreover, the F-F group had a significantly greater mean contact angle than the control group (t_54_ = 3.9, p < 0.001; SE = 6.1; d = 1.1). No significant differences were seen between the HF-F and F-F groups.

### Proportion of contact angles in range

**Fig. 9:**
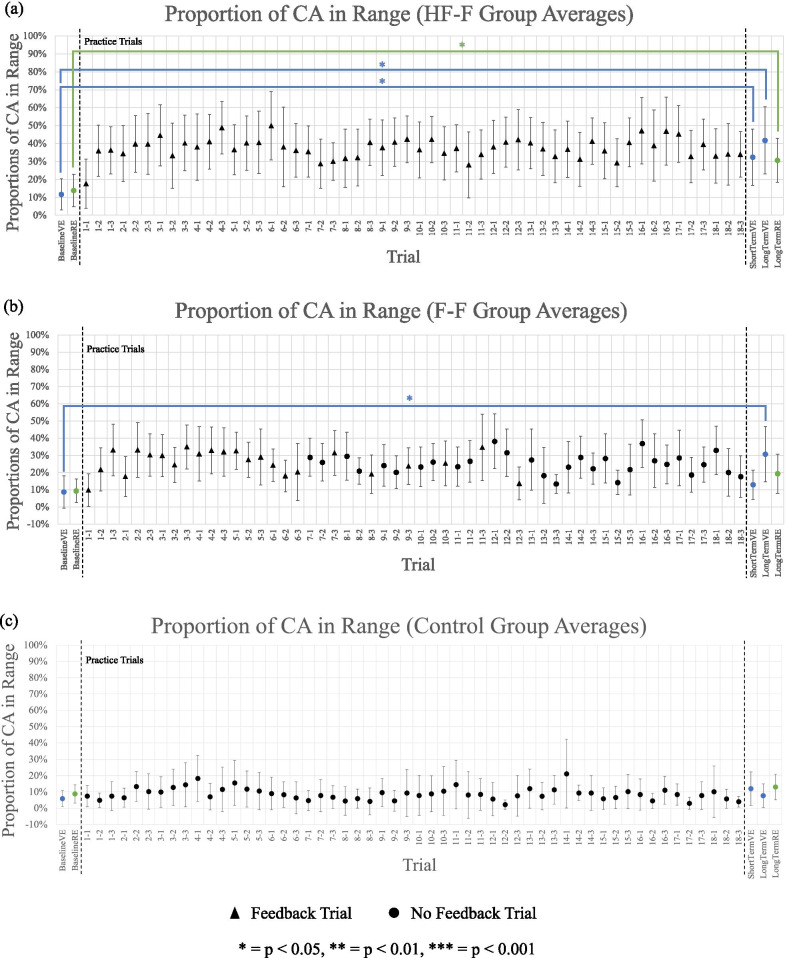
Proportion of contact angles in range (group average) for assessments (in VE and RE) and practice trial sequences for **a** HF-F group, **b** F-F group, and **c** control group. Each practice trial is split into three segments of straight-line, “no obstacle” sections of sidewalk, each separated by a random obstacle. Error bars represent 95% confidence intervals

The proportion of contact angles within the target range (85°–100°) for each propulsion assessment and practice trial sequence is shown in Fig. 9 for the HF-F group (Fig. 9a), the F-F group (Fig. 9b), and the control group (Fig. 9c). In the VE, using Friedman’s test, a significant overall difference was found between baseline, short-term, and long-term assessments in the HF-F group (p < 0.05). A subsequent Wilcoxon signed-rank test determined that in the VE, from baseline to short-term assessment, the HF-F group improved significantly from 12 to 32% (p < 0.05). From baseline to retention assessment in the VE, the HF-F group improved significantly from 12 to 42% (p < 0.05). This group also showed significant improvement in the RE, increasing proportion of angles in range from 14 to 31% (p < 0.05). The Friedman’s test also showed a significant overall difference between baseline, short-term, and long-term assessments for the F-F group in the VE (p < 0.05). The Wilcoxon signed-rank test found that in the from baseline to short-term assessment in the VE, no significant improvements were made by the F-F group. However, this group significantly improved from 8 to 31% in the VE from baseline to long-term retention assessment (p < 0.05) but made no improvement in the RE. Finally, as per the Friedman’s test, there were no significant overall differences between baseline, short-term, and long-term assessments in the control group in the VE. No subsequent Wilcoxon signed-rank test was performed. In the RE, there was also no appreciable change from baseline to long-term assessment.

**Fig. 10:**
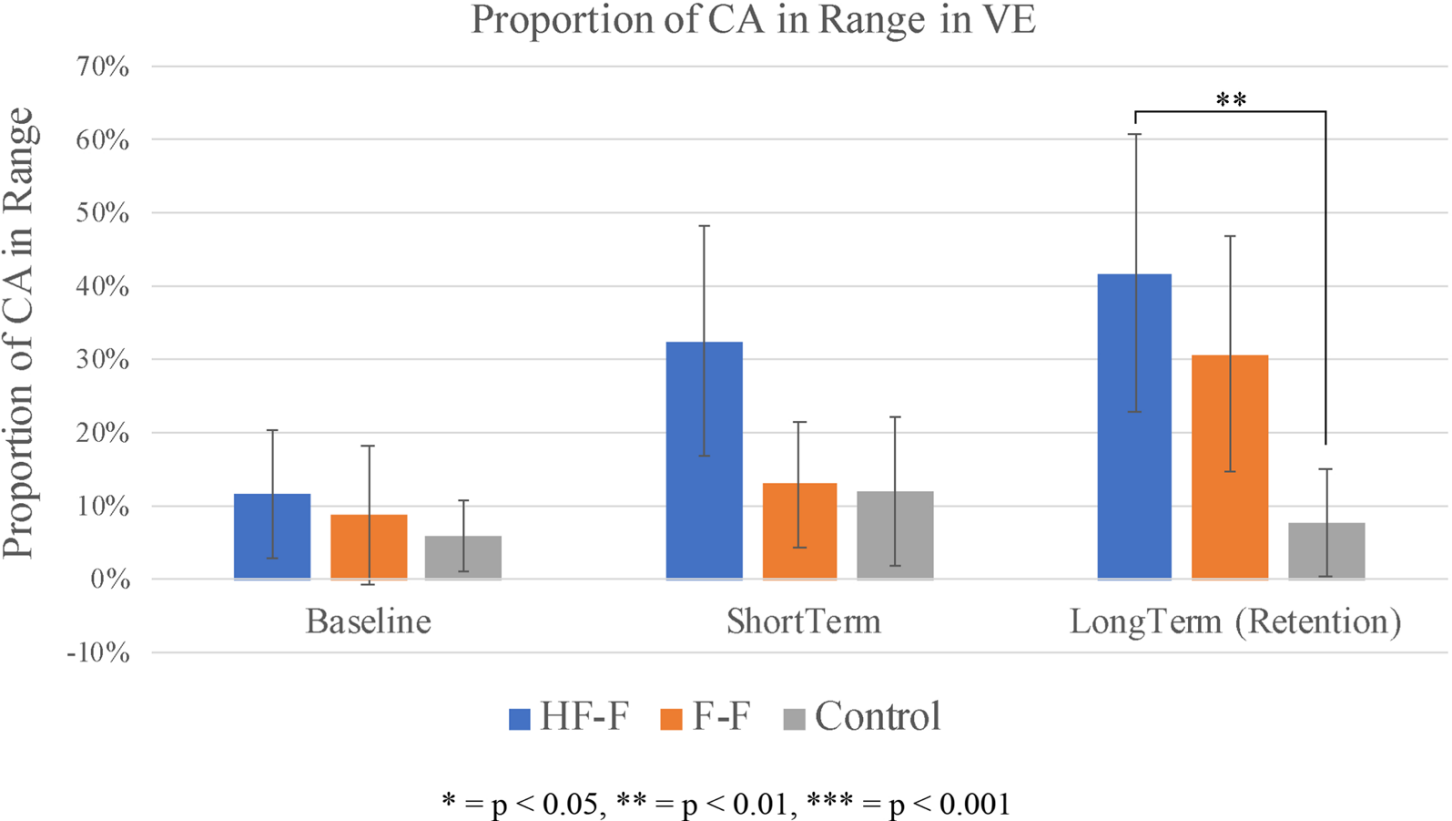
Proportion of contact angles in range (group average) for baseline, short-term, and long-term propulsion assessments in the virtual environment. Error bars represent 95% confidence intervals

Figure 10 displays the proportion of contact angles in range for each group at baseline, short-term, and long-term propulsion assessments in the VE. Using the Kruskal–Wallis test, a significant overall difference was found between groups only at long-term assessment in the VE (p < 0.05). Subsequent Mann–Whitney U tests were then used to compare evaluate differences between groups at long-term assessment in the VE. At long-term retention assessment in the VE, the HF-F group significantly outperformed the control group (p < 0.01). The difference between the F-F and control groups was not significant. The difference between the HF-F and F-F groups was also not statistically significant.

**Fig. 11:**
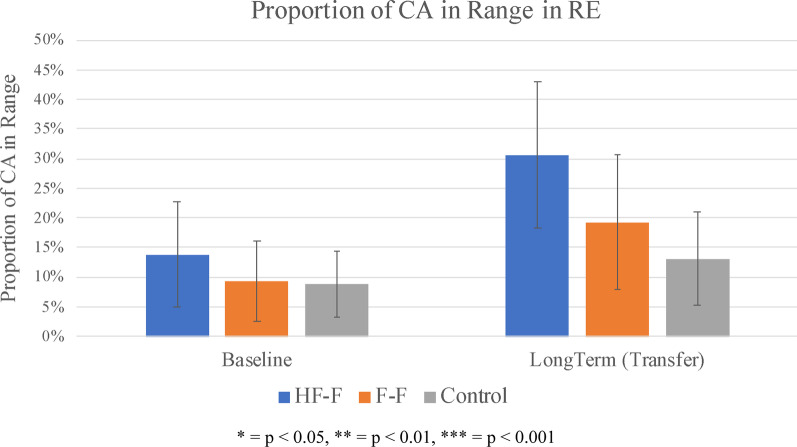
Proportion of contact angles in range (group average) for baseline and long-term propulsion assessments in the real environment. Error bars represent 95% confidence intervals

Figure 11 shows the proportion of contact angles in range for each group at baseline and long-term propulsion assessment in the RE. At long-term transfer assessment, no group differences were seen for this variable.

### Push frequency

Finally, for the measure of push frequency, tests of fixed effects determined that both group (F_2,54_ = 16.4, p < 0.001) and time (F_1,54_ = 41.4, p < 0.001) each had a significant impact on push frequency. However, the effect of environment was not significant. The effect of the interaction between group, time, and environment also had a significant effect (F_7,67.6_ = 2.6, p < 0.05).

**Fig. 12:**
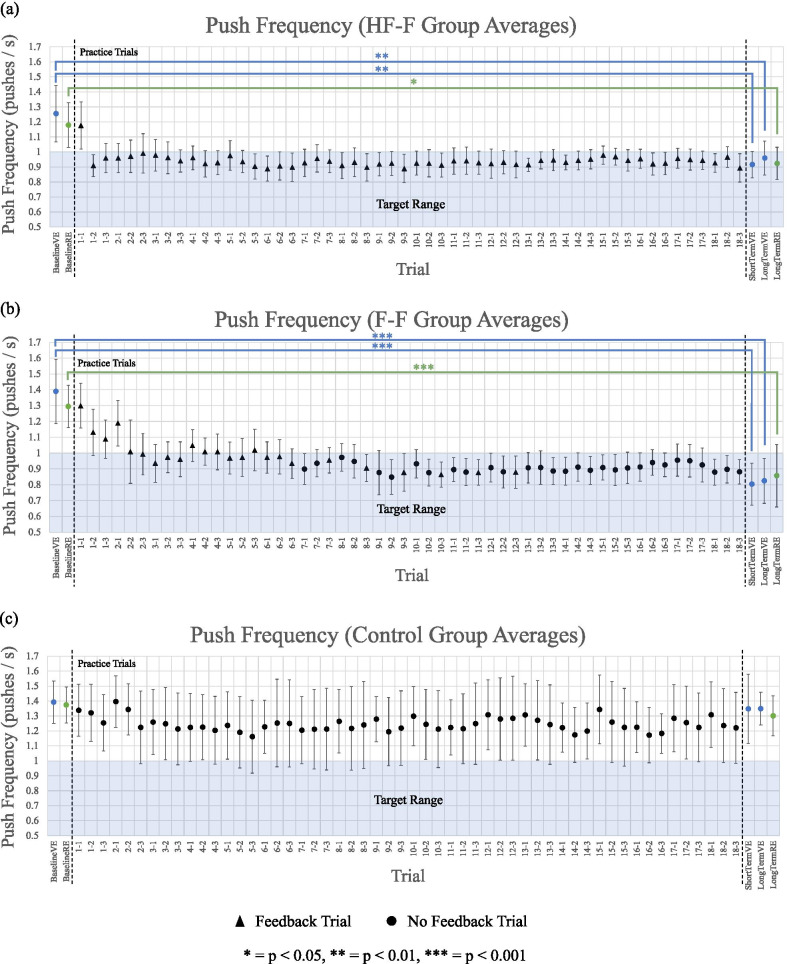
Push frequency (group average) for assessments (in VE and RE) and practice trial sequences for **a** HF-F group, **b** F-F group, and **c** control group. Each practice trial is split into three segments of straight-line, “no obstacle” sections of sidewalk, each separated by a random obstacle. Error bars represent 95% confidence intervals

Group averages of push frequency for each propulsion assessment and practice trial sequence are shown in Fig. 12 for the HF-F group (Fig. 12a), the F-F group (Fig. 12b), and the control group (Fig. 12c). In the VE, from baseline to short-term assessment, the HF-F group significantly improved push frequency from 1.26 to 0.92 pushes/s (t_53.4_ = 2.7, p < 0.01; SE = 0.1; d = 0.8). From baseline to long-term retention assessment in the VE, the HF-F group also significantly improved push frequency from 1.26 to 0.96 pushes/s (t_54_ = 2.8, p < 0.01; SE = 0.1; d = 0.8). In the RE, this group also made significant improvements from 1.18 to 0.92 pushes/s (t_54_ = 2.4, p < 0.05; SE = 0.1; d = 0.7). From baseline to short-term assessment, the F-F group made substantial changes in the VE from 1.39 to 0.80 pushes/s (t_53.4_ = 4.8, p < 0.001; SE = 0.1; d = 1.3). This change was retained, as the F-F group improved push frequency from 1.39 to 0.82 pushes/s (t_54_ = 5.3, p < 0.001; SE = 0.1; d = 1.5) from baseline to long-term retention assessment in the VE. The F-F group also improved in the RE from 1.29 to 0.86 pushes/s (t_54_ = 4.1, p < 0.001; SE = 0.1; d = 1.1). Finally, the control group made no significant changes from baseline to short- or long-term assessments in either environment.

**Fig. 13:**
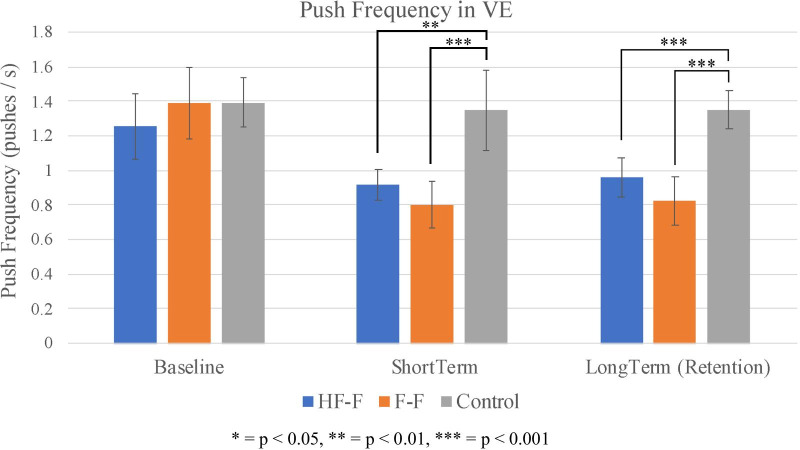
Push frequency (group average) for baseline, short-term, and long-term propulsion assessments in the virtual environment. Error bars represent 95% confidence intervals

The average push frequency of each group at baseline, short-term, and long-term propulsion assessment in the VE is shown in Fig. 13. At short-term assessment, the HF-F group significantly outperformed the control group (t_27_ = 3.7, p < 0.01; SE = 0.1; d = 1.4), as did the F-F group (t_27_ = 4.7, p < 0.001; SE = 0.1; d = 1.8). These changes were maintained. At long-term retention assessment, the HF-F group’s push frequency was significantly lower than that of the control group (t_54_ = 3.9, p < 0.001; SE = 0.1; d = 1.1). The push frequency of the F-F group was also significantly lower than the control group (t_54_ = 5.3, p < 0.001; SE = 0.1; d = 1.4). While the F-F group did produce a lower push frequency than the HF-F group, this difference between the two groups was not significant at either short or long-term assessment.

**Fig. 14:**
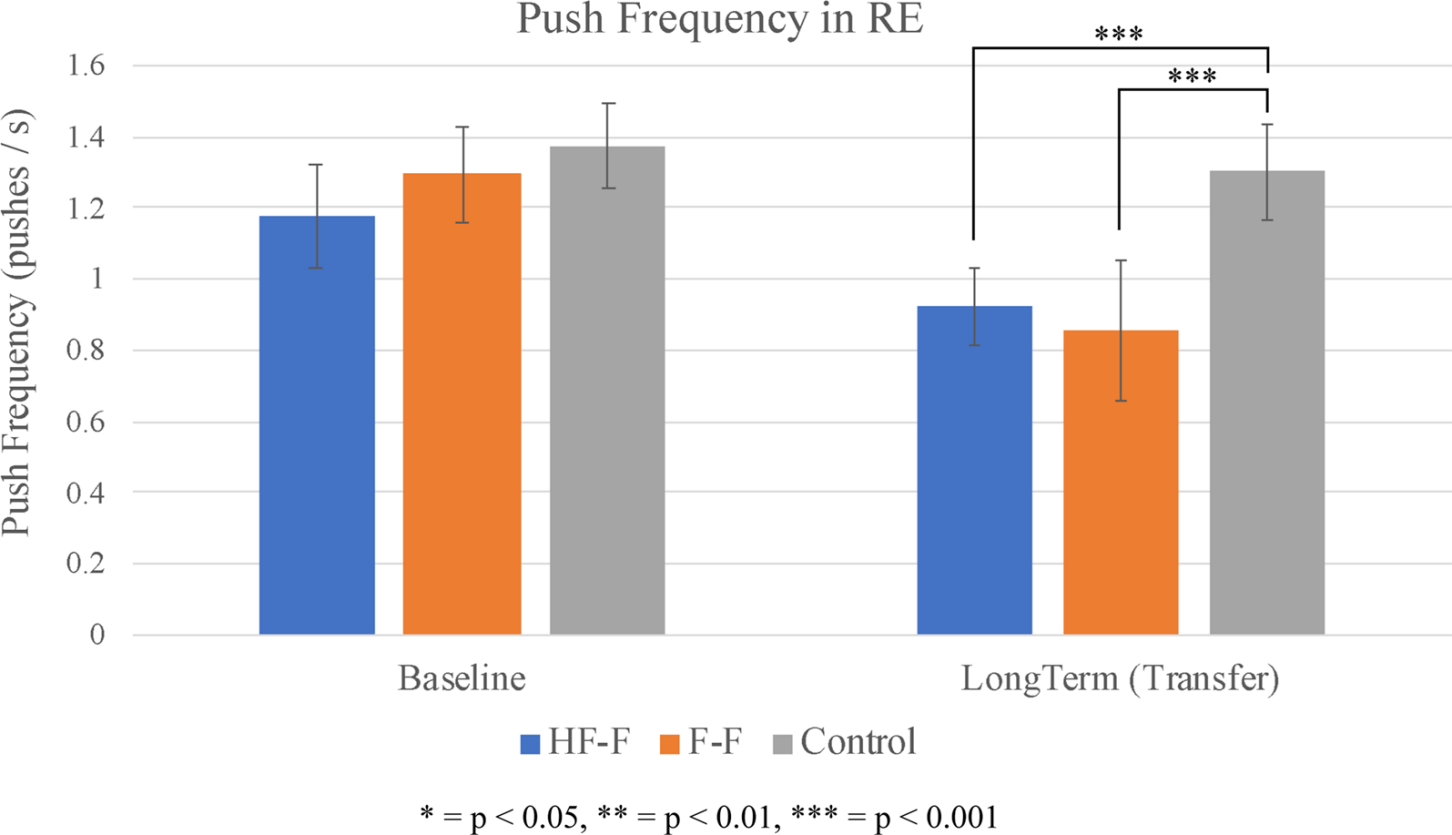
Push frequency (group average) for baseline and long-term propulsion assessments in the real environment. Error bars represent 95% confidence intervals

Figure 14 shows the average push frequency for each group at baseline and long-term assessment in the RE. Similarly to the VE, at 48 h after training, the HF-F group exhibited significantly lower push frequency than the control group (t_54_ = 3.8, p < 0.001; SE = 0.1; d = 1.0) as did the F-F group (t_54_ = 4.5, p < 0.001; SE = 0.1; d = 1.2). No appreciable difference was seen between the HF-F and F-F groups.

## Discussion

The primary objectives of this study were to examine the effects of providing AF during VR propulsion training as well as to test the guidance hypothesis by comparing a faded schedule to a high frequency schedule of AF in this complex task. Additionally, it was critical to assess whether technique learned during simulator training transfers effectively to real world propulsion, as well as to examine each feedback schedule’s effect on transferability of acquired skill.

### Motor learning

To measure the amount of motor learning that took place in each group, propulsion quality during 48-h retention tests were compared to baseline performance. From baseline to retention assessments, participants in both feedback groups successfully increased mean contact angle and proportion of contact angles in range. As well, significant decreases in push frequency were seen for these two intervention groups. These differences before and after training represent positive changes in MWC propulsion technique. Meanwhile, the control group made no appreciable changes in any propulsion outcome measures. These results suggest that the provision of AF about contact angle and push frequency during simulator training is extremely facilitative of both acquisition and retention of this complex skill. This finding is relatively consistent with those from DeGroot et al. [29] who demonstrated that given continuous, real-time visual feedback about contact angle, push frequency, and push force during training, experienced MWC users improved contact angle and push frequency. This study used a population of individuals with mobility limitations which required the use of a MWC with a mean age of 38.6 years. As part of a larger study, wheelchair training took place in three separate sessions approximately 1 week apart, with the effects of treadmill training with AF being evaluated in one of these sessions [29]. Richter et al. [14] also observed improvements in performance when contact angle and push frequency were each individually presented as real-time feedback during training on a wheelchair treadmill. This study also utilized participants who used a MWC as their primary form of transportation. This study also provided continuous real-time feedback which was displayed as a bar graph showing stroke-to-stroke magnitude of a given variable, along with a target magnitude. Changes in propulsion technique from baseline were measured during training with feedback [14]. However, this study did not use long-term retention assessments, assessing performance only during feedback provision.

### Transfer of skill

VR and simulator training offer tremendous benefits such as safety, motivation, and opportunity to control practice as well as provide AF. However, training in such a controlled environment is valuable only if it produces positive change in movement execution in the real-world. In order to determine how effectively learned propulsion technique can transfer to real-world over-ground propulsion, performance on long-term transfer assessments (48 h post-training) was compared to baseline RE performance. Both feedback intervention groups significantly improved the outcome measures of mean contact angle and push frequency following training. However, only the HF-F group increased proportion of contact angles in range. The dramatic improvements seen in both mean contact angle and push frequency demonstrate that given AF during simulator practice, individuals were able to improve mean temporal variables when propelling over-ground. Meanwhile, the control group did not make any significant changes to any outcome measures during over-ground propulsion. The use of transfer assessments in the RE demonstrates that training in the miWe VR simulator is beneficial for developing safe and efficient propulsion technique in the real world, but only when training is augmented with feedback.

### Practice without feedback

In the control group, there was no effect of training on propulsion quality. While this group did not receive any feedback about propulsion biomechanics during practice, they were given instructions about appropriate technique using statements from the clinical practice guidelines prior to training. Additionally, throughout training, all participants were verbally reminded to maintain a semi-circular propulsion pattern. Despite this instruction and guidance, practice without AF did not have a positive effect on mean contact angle, proportion of contact angles in range, or push frequency. Therefore, findings from this study support the contention that practice alone is insufficient for producing positive change in propulsion technique, even when instructions from clinical practice guidelines are provided. However, this inference is in contrast to findings from Rice et al. [30] which imply that instruction about MWC propulsion alone is sufficient for acquisition as well as long-term retention of appropriate technique. This discrepancy in findings may be explained by the type and content of instruction provided. While Rice et al. [30] used an instructional video and slide show, our protocol included only verbal instruction and reminders throughout practice. Further research may investigate specific elements and modality of instruction and guidance for training this specific skill.

Further, our results demonstrate that simply maintaining a semi-circular propulsion pattern does not ensure improvements in contact angle and push frequency measures. Rather, provision of AF about these temporal biomechanical outcomes during practice is absolutely necessary when training novel MWC users.

### Feedback delivery schedule

For the complex skill of MWC propulsion technique, this study found limited evidence for the validity of the guidance hypothesis. Those in the F-F group did improve mean contact angle and push frequency more than the HF-F group in both the VE and RE. However, no significant differences between these two groups were found for any outcome measure. This finding is not consistent with those reported by studies utilizing simple, discrete tasks. Winstein and Schmidt [38] demonstrated that a faded schedule of feedback is significantly advantageous for a task that requires learners to produce a movement pattern with a lever. Winstein et al. [39] also reported that when training individuals to extend a lever to a target position, a faded schedule of AF was beneficial for both skill retention and transfer to similar tasks.

The weak effect of a faded feedback schedule in this study may be explained by the task difficulty and its interaction with AF frequency or delivery schedule. MWC propulsion is a complex and continuous task, requiring coordination of various joints and segments including the hand, wrist, elbow, shoulder, and even trunk. Fujii et al. [55] found that when using a system that emits non-speech audio to provide knowledge of performance regarding a joint coordination pattern task, high relative frequency feedback was more effective at facilitating motor learning. Wulf et al. [56] also reported that when learning the relatively complex motor skill of producing slalom movements on a ski simulator, providing feedback for 100% of trials was more effective than providing such feedback for 50% of trials. This is likely explained by the influence of task difficulty and its interaction with feedback frequency and schedule. It has been suggested that high frequencies of feedback may be more beneficial for learning complex motor skills until a certain level of expertise is reached [56]. It is possible that if given more practice trials to acquire complex tasks, the benefit of a faded schedule of feedback may emerge as observed in simpler tasks. That is, complex tasks may require longer periods of practice, with feedback reduced more gradually.

It is also important to note that following practice, in the VE, the F-F group produced mean contact angle that slightly exceeded the upper bound of the target range which also resulted in a low proportion of contact angles in range. Since there exists both a lower and upper bound to the target range of this variable, more guidance may be required to prevent learners from overshooting contact angle. During initial practice trials, this group’s mean contact angle was well below the target range, hence feedback encouraged learners to increase this measure. Early in practice, most participants receiving feedback were focused primarily on increasing contact angle and decreasing push frequency. However, as AF began to be reduced after six practice trials, it is possible that participants continued to fixate on increasing contact angle, eventually resulting in pushes that exceeded 100°. Without a high frequency of feedback later in practice, these participants may not have had the opportunity to detect this error. In contrast, with continuous high-frequency feedback, when contact angle exceeded this upper bound later in practice, AF would always be shown to direct learners back to the target range for subsequent trials. For feedback variables with both a lower and upper bound, AF may be required for a longer period of time in order to facilitate guidance and error detection for errors of both extremes.

Another explanation for lack of differences found between feedback schedules is that perhaps a longer retention period was required in order reveal the benefit of a faded schedule. Aoyagi et al. [40] reported that the primary benefit of faded feedback scheduling during practice was prolonging retention of skill. With additional delayed retention and transfer tests with even greater retention periods, differences between feedback schedules may be more distinct.

### Study limitations

One limitation to this study is low sample size. Although we doubled the calculated sample size based on findings from previous studies, with more participants in each feedback intervention group, the results may have offered more insight into the effects of different feedback schedules.

Further, this study investigated the effects of providing feedback on contact angle and push frequency only. Propulsion quality was also assessed using these two outcome measures. While AF regarding the temporal variables of contact angle and push frequency appears to be most effective for teaching propulsion technique, future studies could additionally provide AF about kinetic variables and measure these as other indices of propulsion quality [32]. However, as concluded by de Groot et al. (2002), measures of force are more resistant to change, and may require longer periods of practice over several weeks in order to elicit improvements [33]. Additionally, complex cross-variable effects and trade-offs exist between these properties of propulsion dynamics and biomechanics. For example, optimizing the temporal variables of contact angle and push frequency may promote force increase. Richter et al. [14] demonstrated that when experienced MWC users were instructed to minimize push frequency with real-time feedback for this variable, improvements in frequency were achieved with inadvertent increases in peak force. However, when encouraged to decrease push frequency by 10%, peak force increased by a substantially smaller margin. Significant changes in temporal parameters, particularly push frequency, may have severe negative impacts on kinetic parameters such as peak force. Rice et al. [30] also observed improvements in contact angle and push frequency after receiving AF about contact angle, push frequency, and peak force. However, while peak force increased significantly immediately after practice, this increase was not sustained at long-term follow-up assessment. A limitation of the current study is that changes in force were not examined. However, efforts were made to limit the amount of force increase, by providing propulsion instructions from clinical practice guidelines, discouraging maximization of contact angle, and reminding learners to utilize a semi-circular pattern.

Another important limitation to this study was the need to use inexperienced, able-bodied participants as many MWC users may face additional needs and challenges during motor learning and MWC propulsion. Cognitive and psychological impairments such as deficits in attention and memory as well as depression and anxiety may negatively affect motor learning processes [57–61]. Additionally, acquisition of ideal propulsion technique may be hindered in many MWC users due to upper body motor impairments, such as limited trunk control and reduced grip strength [62, 63]. For these reasons, findings from this study may not translate directly to a clinical population of wheelchair users. However, individuals with physical as well as cognitive disabilities also learn skilled movements through motor learning principles [64–66]. Further studies may examine the effects of propulsion training with VR and AF about propulsion biomechanics on clinical populations such as those who have recently suffered traumatic spinal cord injury.

Similarly, this study used participants between the ages of 18–35 as this age range has the highest incidence of traumatic spinal cord injury for both males and females in Canada [67]. However, the mean age of MWC users in Canada is 65.4 years [1]. This represents another important limitation as aging can affect processes associated with motor learning [68–71]. For example, Smith et al. [70] found that motor learning is significantly slower in individuals older than 62 years. However, this study also suggested that some newly acquired fine motor skills were retained for 2 years in adults 18–95 years old and appear to be less affected by age-related changes. Additionally, Berghuis et al. [72] reported that healthy older adults with mean age of 70.9 years are able to learn and retain complex motor skills but have difficulty transferring acquired skills to novel contexts. Future studies may also examine the effects of VR and accompanying AF on motor learning of propulsion in older adults which may be more representative of a population of MWC users in Canada.

## Conclusion

Provision of AF about propulsion biomechanics during MWC training in a VR simulator is critical for retention and transfer of appropriate propulsion technique, which is a complex, ecological motor skill. Further, in accordance with the guidance hypothesis, there is a small but insignificant additional benefit of providing this AF in a faded schedule. These findings should be considered during the design of specific propulsion training programs, as acquisition and maintenance of ideal propulsion technique may mitigate long-term upper extremity pain and injury in MWC users.

## Data Availability

The datasets used in the current study are available from the corresponding author on reasonable request.

## Abbreviations

AF: Augmented feedback
F-F: Faded feedback
HF: High frequency
HF-F: High frequency feedback
LF: Low frequency
miWe: McGill Immersive Wheelchair Simulator
MoCA: Montreal Cognitive Assessment
MWC: Manual wheelchair
NF: No feedback
RE: Real environment
VE: Virtual environment
VR: Virtual reality
